# Large-Scale Plasma Analysis Revealed New Mechanisms and Molecules Associated with the Host Response to SARS-CoV-2

**DOI:** 10.3390/ijms21228623

**Published:** 2020-11-16

**Authors:** Elettra Barberis, Sara Timo, Elia Amede, Virginia V. Vanella, Chiara Puricelli, Giuseppe Cappellano, Davide Raineri, Micol G. Cittone, Eleonora Rizzi, Anita R. Pedrinelli, Veronica Vassia, Francesco G. Casciaro, Simona Priora, Ilaria Nerici, Alessandra Galbiati, Eyal Hayden, Marco Falasca, Rosanna Vaschetto, Pier Paolo Sainaghi, Umberto Dianzani, Roberta Rolla, Annalisa Chiocchetti, Gianluca Baldanzi, Emilio Marengo, Marcello Manfredi

**Affiliations:** 1 Department of Translational Medicine, University of Piemonte Orientale, 28100 Novara, Italy; elettra.barberis@uniupo.it (E.B.); elia.amede@uniupo.it (E.A.); virginia.vanella@uniupo.it (V.V.V.); rosanna.vaschetto@med.uniupo.it (R.V.); gianluca.baldanzi@med.uniupo.it (G.B.); 2 Center for Translational Research on Autoimmune and Allergic Diseases, University of Piemonte Orientale, 28100 Novara, Italy; sara.timo@uniupo.it (S.T.); giuseppe.cappellano@med.uniupo.it (G.C.); davide.raineri@uniupo.it (D.R.); annalisa.chiocchetti@med.uniupo.it (A.C.); emilio.marengo@uniupo.it (E.M.); 3 Department of Sciences and Technological Innovation, University of Piemonte Orientale, 28100 Alessandria, Italy; 4 Department of Health Sciences, University of Piemonte Orientale, 28100 Novara, Italy; 20032501@studenti.uniupo.it (C.P.); umberto.dianzani@med.uniupo.it (U.D.); roberta.rolla@med.uniupo.it (R.R.); 5 Internal and Emergency Medicine Departments, Department of Translational Medicine, University of Piemonte Orientale, 28100 Novara, Italy; micolcittone@gmail.com (M.G.C.); eleonora.rizzi91@yahoo.it (E.R.); anitapedrinelli@yahoo.it (A.R.P.); veronica.vassia@gmail.com (V.V.); casciaro90@yahoo.it (F.G.C.); simonasimop@alice.it (S.P.); ila.nerici@gmail.com (I.N.); alessandra.galbiati92@gmail.com (A.G.); eyal.hayden@maggioreosp.novara.it (E.H.); pierpaolo.sainaghi@med.uniupo.it (P.P.S.); 6 Azienda Ospedaliero-Universitaria “Maggiore della Carità”, 28100 Novara, Italy; 7 Metabolic Signalling Group, School of Pharmacy & Biomedical Sciences, Curtin University, Perth 6102, Australia; marco.falasca@curtin.edu.au

**Keywords:** SARS-CoV-2, biomarkers, metabolism, fatty acids

## Abstract

The novel severe acute respiratory syndrome coronavirus 2 (SARS-CoV-2) has spread to nearly every continent, registering over 1,250,000 deaths worldwide. The effects of SARS-CoV-2 on host targets remains largely limited, hampering our understanding of Coronavirus Disease 2019 (COVID-19) pathogenesis and the development of therapeutic strategies. The present study used a comprehensive untargeted metabolomic and lipidomic approach to capture the host response to SARS-CoV-2 infection. We found that several circulating lipids acted as potential biomarkers, such as phosphatidylcholine 14:0_22:6 (area under the curve (AUC) = 0.96), phosphatidylcholine 16:1_22:6 (AUC = 0.97), and phosphatidylethanolamine 18:1_20:4 (AUC = 0.94). Furthermore, triglycerides and free fatty acids, especially arachidonic acid (AUC = 0.99) and oleic acid (AUC = 0.98), were well correlated to the severity of the disease. An untargeted analysis of non-critical COVID-19 patients identified a strong alteration of lipids and a perturbation of phenylalanine, tyrosine and tryptophan biosynthesis, phenylalanine metabolism, aminoacyl-tRNA degradation, arachidonic acid metabolism, and the tricarboxylic acid (TCA) cycle. The severity of the disease was characterized by the activation of gluconeogenesis and the metabolism of porphyrins, which play a crucial role in the progress of the infection. In addition, our study provided further evidence for considering phospholipase A2 (PLA2) activity as a potential key factor in the pathogenesis of COVID-19 and a possible therapeutic target. To date, the present study provides the largest untargeted metabolomics and lipidomics analysis of plasma from COVID-19 patients and control groups, identifying new mechanisms associated with the host response to COVID-19, potential plasma biomarkers, and therapeutic targets.

## 1. Introduction

The novel coronavirus (SARS-CoV-2) led to an outbreak of viral pneumonia in Wuhan, China, which began in December 2019 [1]. The virus has spread to nearly every continent, and case numbers continue to rise, registering over 1,250,000 deaths worldwide [2]. Patients have clinical manifestations, including fever, cough, shortness of breath, muscle ache, confusion, headache, sore throat, rhinorrhea, chest pain, diarrhea, nausea, and vomiting [3,4]. The main organ targeted by SARS-CoV-2 is the low respiratory tract, and some patients develop a life-threatening acute respiratory distress syndrome, although acute myocardial injury and chronic damage to the cardiovascular system have also been found [5]. Although more than 80% of COVID-19 patients experience only mild symptoms, conditions can rapidly progress from mild to severe, particularly in the absence of adequate medical care.

To date, most SARS-CoV-2 studies have focused on its epidemiological and clinical characteristics [6,7]. Recent studies demonstrated that SARS-CoV-2 binds to the angiotensin converting enzyme 2 (ACE2) on the cell surface [8] and that the virus dramatically impacts the immune function [9]. However, the physiological changes associated with SARS-CoV-2 are not yet clear.

Profiling the human bio-fluids, proteome, lipidome, and metabolome, provides insight into critical illness, as well as diseased states, as all these molecules play essential roles in pathogenesis and in several cell functions. Longitudinal analyses of molecular components can be used not only to improve the knowledge of diseases, but also for precision health care. Previous studies have demonstrated significant alterations of metabolome and lipidome in human plasma caused by viral infections, such as the Ebola virus disease [10,11]. In addition, metabolomics analysis has been successfully used to identify potential biomarkers for the diagnosis and prognosis of H1N1 influenza pneumonia [12] and to identify lipid metabolism alterations in recovered severe acute respiratory syndrome (SARS) patients after 12 years of infection [13]. The analysis of plasma lipids from Ebola virus disease patients revealed critical illness and disease states as lipids play essential roles as membrane structural components, signaling molecules, and energy sources [11].

Recently, a targeted metabolomics and lipidomics approach identified alterations related to the course of disease in COVID-19 patients, indicating an altered energy metabolism and hepatic dysfunction. However, the study involved a limited number of Chinese patients (34) and used a targeted approach focused on a well-defined number of molecules [14]. Another study [15] identified molecular changes in the sera of COVID-19 patients, implicating the dysregulation of macrophages, platelet degranulation, complement system pathways, and massive metabolic suppression. While the study involved proteomics and metabolomics, the research was limited to 46 patients from a Chinese hospital. Meanwhile, Song et al. identified metabolic dysregulation and a positive correlation between monosialodihexosyl gangliosides (GM3s)-enriched exosomes and COVID-19 pathogenesis, although only 50 Chinese patients were involved in the study [16]. Alterations of the kynurenine pathway and fatty acid metabolism were found in a small cohort of 33 COVID-19 patients in the United States compared to a young control group (33-year-olds) [17].

Thus, our knowledge of the effects of SARS-CoV-2 on host targets remains largely limited, hampering our understanding of COVID-19 pathogenesis and the development of therapeutic strategies. In particular, there has not been a comprehensive and untargeted metabolomics and lipidomics study on European patients affected by COVID-19. This focus area is very relevant, particularly since recent research found that a mutated coronavirus showed a significant boost in infectivity and that different mutations of the virus are present [18]. Moreover, the limited numbers of patients involved in the previously cited studies [14,15,16,17] point out the necessity of conducting a larger and more representative research study.

As part of the recently formed COVID-19 mass spectrometry coalition (www.covid19-msc.org) [19], the present study provides the first largest untargeted metabolomics and lipidomics analysis of plasma from COVID-19 patients and control groups. The samples come from northern Italy, the Italian epicenter of the pandemic. Italy was the first western country to experience the COVID-19 disease. Our findings show that there are several mechanisms and pathways associated with the host response to SARS-CoV-2, together with some interesting potential biomarkers and therapeutic targets.

## 2. Results

### 2.1. Untargeted Lipidomics and Metabolomics Profiling of COVID-19 Plasma

**Figure 1:**
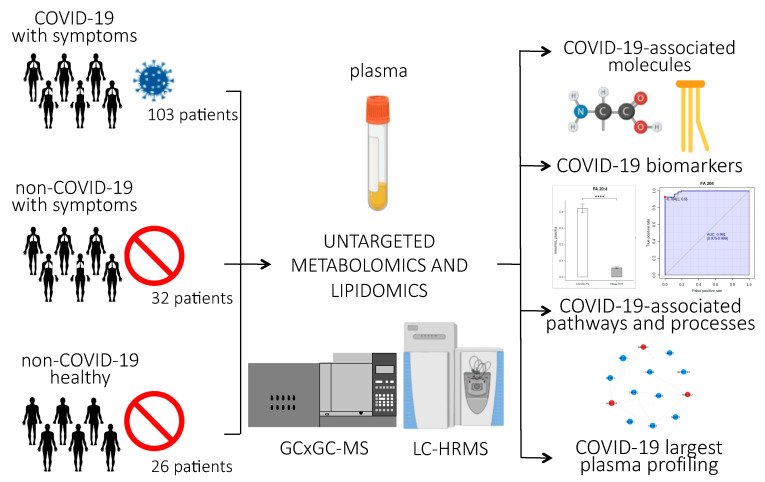
Experimental design of the study: Untargeted lipidomics and metabolomics analyses were performed on plasma samples from 103 patients infected with SARS-CoV-2, 84 of whom had non-critical COVID-19, while 19 had critical COVID-19 and recovered in the ICU; 20 non-COVID-19 patients with similar clinical symptoms as the COVID-19 patients; 26 healthy subjects; and 12 ICU patients who tested negative for COVID-19. The abundance of small molecules and lipids were used to identify COVID-19-associated biomarkers, pathways, and processes related to the host response to the virus. (*p*-value < 0.0001 = ****).

Figure 1 provides an overview of the experimental design of the study. Briefly, untargeted lipidomics and metabolomics analyses were performed on plasma samples of 161 subjects, 103 of whom were infected with SARS-CoV-2. Clinical characteristics of the patients are reported in Table 1. The patients involved in the study were from northern Italy, the Italian epicenter of the pandemic, in particular from the city of Novara. We considered critical patients as those with respiratory failure admitted to the intensive care unit requiring mechanical ventilation (ICU-COVID-19), and non-critical patients as all other patients with mild to severe respiratory failure requiring oxygen supplementation but neither mechanical nor non-invasive ventilation. Out of the 103 COVID-19 patients enrolled, 19 were critical and 84 non-critical. Out of 32 patients admitted for pneumonia and/or respiratory failure with negative nucleic acid test results, 20 were non-critical and 12 critical. Twenty-six healthy subjects were enrolled as control. For the lipidomic analyses, we used an ultra-performance liquid chromatography/tandem mass spectrometry (UPLC-MS/MS) untargeted approach to analyze the plasma samples, while untargeted metabolomics analysis was performed using a bi-dimensional gas chromatography/mass spectrometer (GCxGC-MS).

Lipidomics analysis was performed using both positive and negative ionization to cover various lipid classes. The acquired data was processed using MS-DIAL for automated mass spectral deconvolution of high-resolution MS data [20] followed by a MS/MS library search using LipidBlast. The analysis led to the detection of 2075 and 1108 identified and unidentified lipid species in positive and negative modes, respectively, while the total numbers of identified lipids were 467 and 89 (Table S1). The lipid species belonged to the following lipid classes: acylcarnitines (CAR), cholesteryl esters (CEs), diacylglycerols (DGs), triacylglycerols (TGs), non-esterified fatty acids (FAs), phosphatidylinositols (PIs), lysophosphatidylcholines (LPCs), lysophosphatidylethanolamines (LPEs), phosphatidylcholines (PCs), phosphatidylethanolamines (PEs), and sphingomyelins (SMs). The estimated concentrations of all lipids (expressed as nmol/mL of plasma) were calculated.

The reproducibility of the data was evaluated using spiked standards and quality control (QC) samples. Relative standard deviations (RSDs) for spiked standards in QC samples were lower than 20% for almost all 10 standards used (Table S2). No sensitivity loss across the analyses was observed, and the retention time reproducibility was good (<1%). The principal component analysis (PCA) score plot showed that the QC replicates were well clustered together, reflecting the instrumental reproducibility, as reported in Figure S1A,B. For the metabolomics analysis, the untargeted GCxGC-MS approach allowed for the quantification of more than 500 small molecules. These molecules included amino acids; short, medium, and long-chain fatty acids; sugars; organic acids; and steroids. The similarity threshold for analyte identification was set to 750, and all mass spectra and the retention index were manually checked and compared to the NIST library and the literature. The curated results identified the presence of 253 relevant analytes (Table S3); the relative abundance of all analytes was obtained for each sample.

Spiked standards and QC samples were used to evaluate the reproducibility of the sample preparation and the analysis. The RSDs for the spiked standards were 8% and 15%, while they were lower than 30% for the other selected molecules in the QC samples (Table S4). While the reproducibility of the retention time was very good, we observed a small loss of sensitivity across the analysis that was corrected using QC samples and internal standards. The PCA score plot shows that the QC replicates were well clustered together, reflecting the instrumental reproducibility, as reported in Figure S1C.

### 2.2. Molecules Alterations in COVID-19 Plasma

The samples were first analyzed using multivariate analysis. We grouped non-critical positive COVID-19 patients and healthy subjects using a PCA score plot. The PCA provided a clear separation of samples according to the diagnosis for the lipidomics and metabolomics profiles (Figure S2). To confirm the PCA results, the samples were also grouped using a supervised partial least square discriminant analysis (PLS-DA), which was calculated to achieve maximum separation between the two groups of patients (Figure S3). The most predictive or discriminative features that are potentially useful in helping sample classification were also reported through the VIP (variable of importance in projection) score. The VIP score summarized the most prominent molecules contributing to the observed phenotypic variations in the COVID-19 plasma (Figure S3D–F). Lipidomics and metabolomics differences between the two groups of samples were mostly due to several species of PCs, PEs, LPCs, and TGs, as well as sugars and fatty acids. 

**Figure 2:**
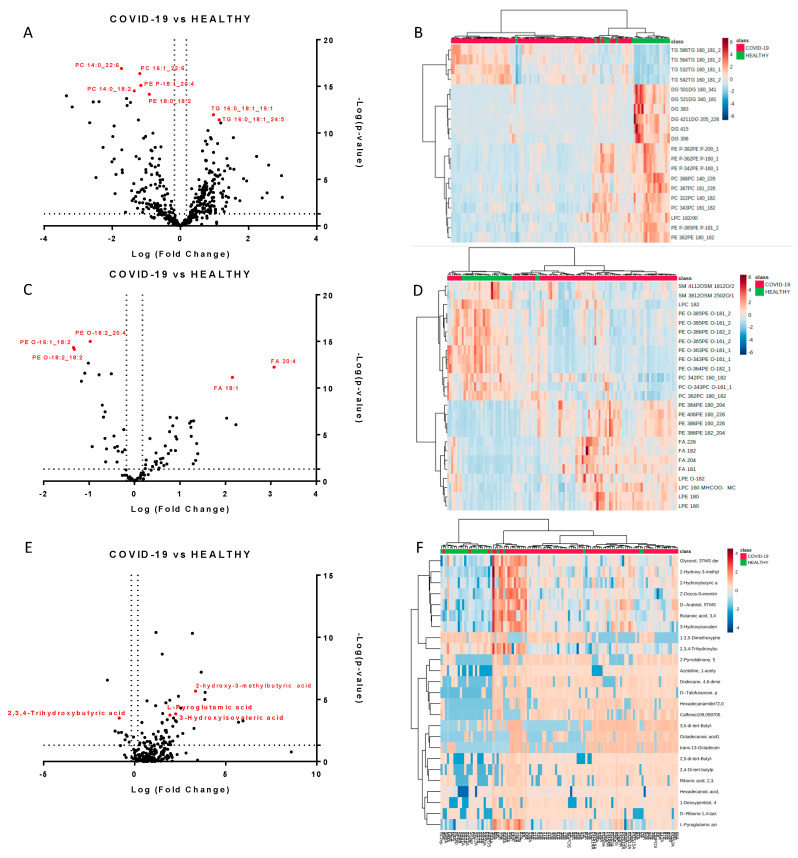
Modulated lipids and small molecules in SARS-CoV-2 infection. Volcano plots of quantified lipids in positive (**A**) and negative (**C**) modes. A total of 265 lipids were modulated with a *p*-value < 0.05 and a fold change > 1.5. Hierarchical heat maps of quantified lipids in positive (**B**) and negative (**D**) modes, highlighting the two clusters of samples, with COVID-19 patients in red and healthy subjects in green. Panels (**E**,**F**) report the volcano plot of the quantified small molecules and the heat map, respectively.

We next performed a univariate analysis of quantified lipids and metabolites using MetaboAnalyst software. No data filtering was applied during the processing, and log transformation and auto-scaling normalization were used to preprocess the data. A total of 265 lipids were modulated in plasma from COVID-19 patients (*p*-value < 0.05, fold change > 1.5). The volcano plots (Figure 2A,C) showed the most significant differences among the lipids and the positive or negative fold-changes in the COVID-19 patients compared to the healthy group. To summarize the univariate results, we used a heat map (Figure 2B,D) to display the fold changes of the top modulated lipids. The heat map allowed for the visualization of two clusters of samples and different lipid levels. The complete list of modulated lipids is reported in Table S1.

The monovariate analysis performed on the relative abundance of metabolites allowed for the identification of 75 modulated metabolites (*p*-value < 0.05, fold change > 1.3). Table S3 reports the complete list of modulated molecules together with the fold changes and p-values. The volcano plot and the heat map of the top modulated molecules are reported in Figure 2E,F.

### 2.3. Lipids Are Strongly Involved in the Host Response to COVID-19

**Figure 3:**
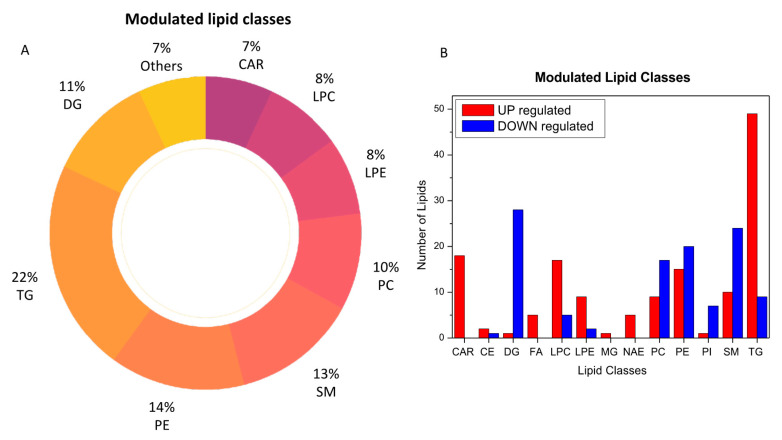
Modulated lipid classes caused by SARS-CoV-2 infection (**A**) and the number of upregulated (red) and downregulated (blue) lipid species within each single class of lipids (**B**).

The analysis of plasma metabolic changes may reflect new or known mechanisms associated with the host response to SARS-CoV-2 infection. To this aim, we performed bioinformatic analysis to have a better perspective on how metabolic changes detected in blood samples might be due to the overall response and to help identify the potential biochemical links between the SARS-CoV-2 infection and metabolic variations in blood. By comparing the COVID-19 patients with the healthy subjects, we determined that acylcarnitines, diacylglycerols, fatty acids, glycerophosphoethanolamines (LPEs, PEs), glycerophosphocholines (LPCs, PCs), sphingomyelins, and triacylglycerols were the most regulated lipid classes and subclasses (Figure 3).

**Figure 4:**
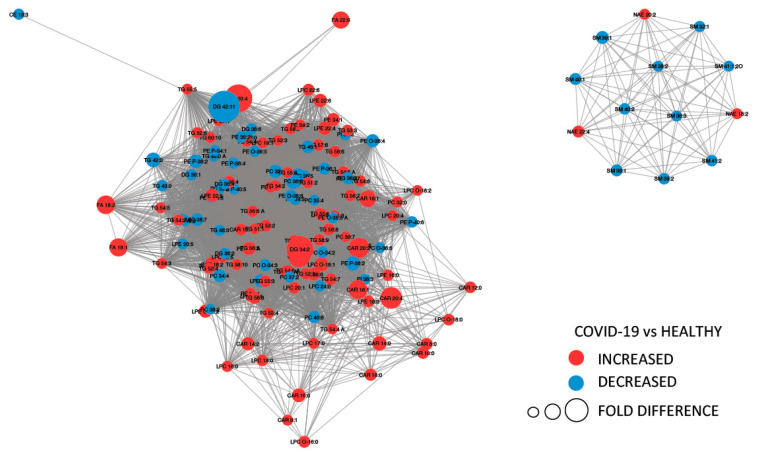
MetaMapp visualization of lipidomic changes in COVID-19 patients. Lipids with increased concentration are depicted using red nodes, while lipids with decreased concentration are represented by blue nodes. The lipids grouped on the right are sphingomyelins and *N*-acyl ethanolamine, while on the left are reported glycerolipids.

Figure 4 provides a MetaMapp visualization of lipidomic changes in COVID-19 patients. Red nodes represent lipids with increased concentration, while blue nodes represent lipids with decreased concentration. PCs were significantly downregulated (65.4%) as a consequence of the infection. The levels of PC (14:0_22:6), PC (16:1_22:6), PC (14:0_18:2), and PC (O-16:1_18:2) were the most decreased, with fold changes of 0.305, 0.443, 0.395, and 0.495, respectively. We also observed that the 57.1% of the glycerophosphoethanolamines were downregulated, while several free fatty acids were upregulated (FA 18:1, FA 18:2, FA 22:6, FA 44:5, and FA 20:4).

Moreover, higher lysophosphatidylethanolamine and lysophophatidylcholine levels were identified. This can be linked to viral replication in infected cells; the alteration in the lipid homeostasis of host cells is a viral strategy for creating a proper environment for replication. Our data also shows that infected patients had higher levels of all 18 acylcarnitines. Acylcarnitines, which are acyl esters of carnitine, are essential compounds for fatty acid oxidation (FAO) and organic acid metabolism [21].

Furthermore, the differentiating lipids were subjected to KEGG functional enrichment analysis using LIPEA web tool (2018), and we obtained two significant pathways as a result: fat digestion and adsorption and glycerophospholipid metabolism, both with Benjamini and Bonferroni corrected *p*-values of less than 0.05.

### 2.4. Amino Acids, Fatty Acids, and the Tricarboxylic Acid (TCA) Cycle Are Involved in the Host Response to SARS-CoV-2 Infection

**Figure 5:**
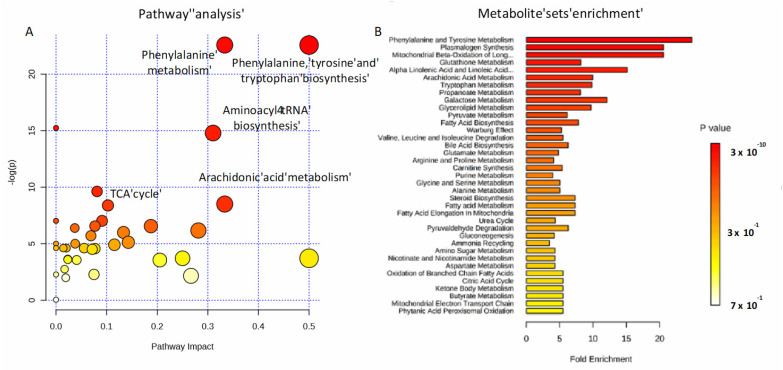
Pathways involved in the infection. Metabolic pathway analysis performed on modulated metabolites (**A**) and metabolite sets enrichment (**B**). Amino-acids, fatty acids, and the TCA cycle are mainly involved during non-critical infection.

To investigate the potential metabolic disruptions in recovered COVID-19 patients, alterations in metabolite abundance were further subjected to metabolic pathways and enrichment analysis based on MetaboAnalyst 4.0 (Figure 5). Modulated metabolites cover several biochemical pathways, which may reveal the metabolic mechanisms of the host response to infection. Phenylalanine, tyrosine and tryptophan biosynthesis, phenylalanine metabolism, aminoacyl-tRNA, arachidonic acid metabolism, and the tricarboxylic acid (TCA) cycle (Figure 5A) were the most important metabolic pathways affected by the SARS-CoV-2 infection. The metabolite set enrichment analysis shown in Figure 5B indicates that phenylalanine and tyrosine metabolism was the metabolite concentrated set most altered by the virus, confirming that amino acid metabolism is a key factor in the non-critical infection.

An interesting distribution of modulated metabolites was observed in the plasma samples of the COVID-19 patients. Higher levels of 2-hydroxy-3-methylbutyric acid, 3-hydroxyisovaleric acid, 2-hydroxybutyric acid, palmitic acid, pyroglutamic acid, succinic acid (butanedioic acid), myristic acid, heptanoic acid, and galactopyranose were detected, while lower levels of 2,3,4-trihydroxybutyric acid and l-valine were found. Two of these metabolites, pyroglutamic and 2,3,4-trihydroxybutyric acid, have already been identified as significantly altered in the serum of patients who recovered from SARS [13]. Biosynthesis of the unsaturated fatty acid pathway, which is related to arachidonic, oleic, palmitic, and stearic acids, was shown to be upregulated in infected patients.

Another important pathway involved in the SARS-CoV-2 infection is aminoacyl-tRNA biosynthesis. This pathway plays an important role in responding to danger signals and regulates immunity against viral infections [22]. Interestingly, the TCA cycle resulted in a significantly modulated consequence of fumaric and succinic acid regulation. Butanoate metabolism, which is related to short chain fatty acid upregulation, was also involved.

### 2.5. Lipidomics and Metabolomics Alterations in Critical COVID-19 Patients

We further investigated the levels of plasma lipids and small molecules in critical COVID-19 patients who were admitted at the intensive care unit (ICU-COVID-19). We first compared 19 ICU-COVID-19 patients to 12 ICU non-COVID-19 patients. We found a significant modulation of 77 lipids and 32 small molecules (Tables S1 and S3). The two groups of samples were well separated using both heat map representations and PCA (Figure S4). Almost all of the upregulated lipids in ICU-COVID-19 patients were triglycerides (Figure S5), and TG 16:1_16:1_18:2, TG 16:0_18:1_24:1, and TG 24:0_18:1_18:1 were the most expressed with fold changes of 14.9, 4.3, and 4.2, respectively. 

Interestingly, we found that the abundance of FA 18:1 and FA 20:4, namely oleic acid and arachidonic acid, was directly correlated to the severity of the disease (Figure S6), with higher levels in ICU-COVID-19 patients. Additional results emerged from the pathway analysis of the small molecule modulations. The enrichment analysis identified a dysregulation of porphyrin metabolism, methionine metabolism, and bile acid biosynthesis (Figure S7). 

We then compared non-critical COVID-19 patients to critical COVID-19 patients (Figure S8). Triglycerides resulted in upregulation in critical patients, confirming the physiological response to critical illness associated with the lipolysis of adipose tissue. Glycerophosphocholine and sphingomyelins were downregulated in critical patients. In addition, the analysis of the small molecules showed a strong involvement of the metabolism of glyoxylate and dicarboxylate, which are responsible for energy metabolic dysfunction (Figure S9). Pathway enrichment analysis also pointed out a significant contribution of gluconeogenesis, which is necessary to maintain blood glucose levels [23]. 

### 2.6. Potential Biomarkers of COVID-19

**Figure 6:**
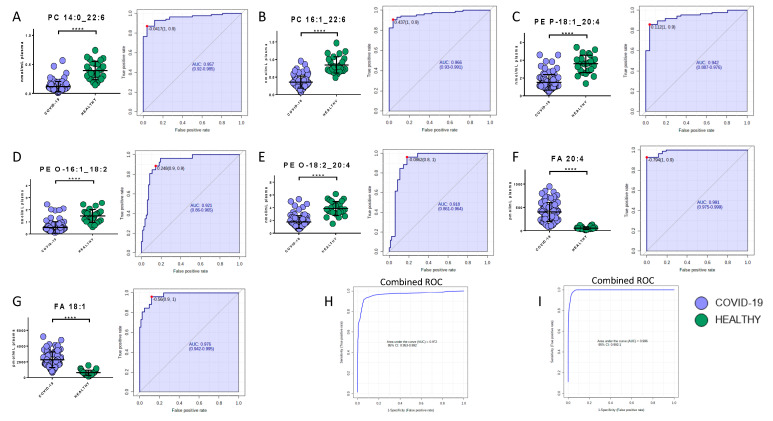
Bar plots (average ± SD) with relative statistical significance (*p*-value < 0.0001 = ****) and ROC curves with the optimal cutoff calculated for each ROC analysis (red dot) are reported in order to show the best potential biomarkers identified using lipidomics analysis. Phosphatidylcholine 14:0_22:6 (**A**), phosphatidylcholine 16:1_22:6 (**B**), phosphatidylethanolamine 18:1_20:4 (**C**), arachidonic acid (**D**), oleic acid (**E**), glycerophosphoethanolamines PE (O-18:2_20:4) (**F**), and glycerophosphoethanolamines PE (O-16:1_18:2) (**G**). Combined ROCs (**H**,**I**) are also shown.

Potential biomarkers were explored by carefully analyzing modulated molecules distribution and by using ROC curves. The analysis reported the presence of seven lipids and eight metabolites able to discriminate COVID-19 patients from healthy subjects. Regarding the lipids quantified in the positive mode, phosphatidylcholine 14:0_22:6, phosphatidylcholine 16:1_22:6, and phosphatidylethanolamine 18:1_20:4 showed area under the curve (AUC) values of 0.96 (sensitivity (SE): 93%, specificity (SP): 89%), 0.97 (SE: 90%, SP: 96%), and 0.94 (SE: 86%, SP: 96%), respectively, while their combined ROC reported an AUC of 0.97 (Figure 6A–C,H). The quantification in the negative mode identified AUC values of 0.99 (SE: 93%, SP: 100%), 0.98 (SE: 96%, SP: 88%), 0.92 (SE: 89%, SP: 86%), and 0.92 (SE: 96%, SP: 82%) for arachidonic acid (FA 20:4), oleic acid (FA 18:1), glycerophosphoethanolamines PE (O-18:2_20:4), and glycerophosphoethanolamines PE (O-16:1_18:2), respectively, while their combined ROC reported an AUC of 1 (Figure 6D–G,I). 

**Figure 7:**
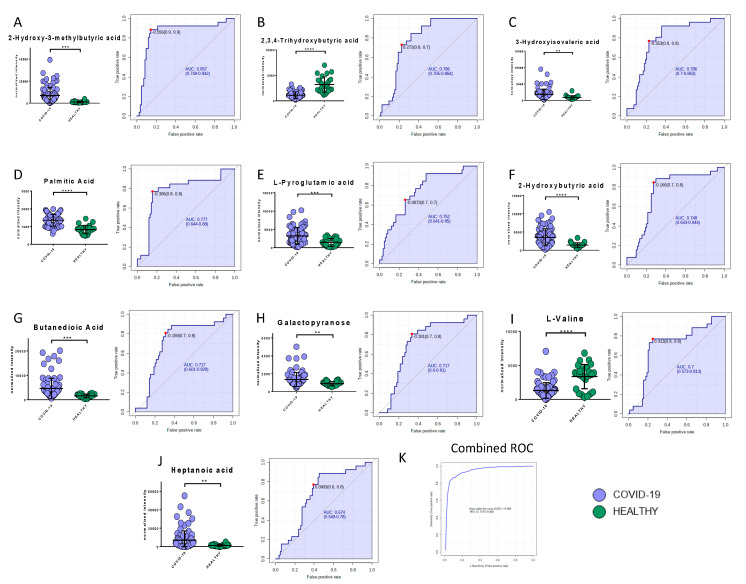
Bar plots (average ± SD) with relative statistical significance (*p*-value < 0.01 = **; *p*-value < 0.001 = ***; *p*-value < 0.0001 = ****) and ROC curves with the optimal cutoff calculated for each ROC analysis (red dot) are reported in order to show the best potential biomarkers identified using metabolomics analysis: 2-hydroxy-3-methylbutyric acid (**A**), 2,3,4-trihydroxybutyric acid (**B**), 3-hydroxyisovaleric acid (**C**), palmitic acid (**D**), L-pyroglutammic acid (**E**), 2-hydroxybutyric acid (**F**), butanedioic acid (**G**), galactopyranose (**H**), l-valine (**I**), and heptanoic acid (**J**). The combined ROC of the best three molecules (2-hydroxy-3-methylbutyric acid, 2,3,4-trihydroxybutyric acid, and 3-hydroxyisovaleric acid) is also shown (**K**).

Among the metabolites, the following biomarkers had the best diagnostic performances: 2-hydroxy-3-methylbutyric acid (AUC = 0.86, SE: 89%, SP: 86%), 2,3,4-trihydroxybutyric acid (AUC = 0.79, SE: 73%, SP: 77%), 3-hydroxyisovaleric acid (AUC = 0.79, SE: 77%, SP: 77%), palmitic acid (AUC = 0.78, SE: 77%, SP: 85%), L-pyroglutammic acid (AUC = 0.75, SE: 65%, SP: 74%), 2-hydroxybutyric acid (AUC = 0.75, SE: 89%, SP: 69%), butanedioic acid (AUC = 0.72, SE: 81%, SP: 69%), galactopyranose (AUC = 0.72, SE: 81%, SP: 67%), myristic acid (AUC = 0.70, SE: 63%, SP: 68%), l-valine (AUC = 0.70, SE: 77%, SP: 75%), and heptanoic acid (AUC = 0.67, SE: 87%, SP: 61%) (Figure 7A–J). The combined ROC of the best three molecules (2-hydroxy-3-methylbutyric acid, 2,3,4-trihydroxybutyric acid, and 3-hydroxyisovaleric acid) reported an AUC of 0.95 (Figure 7K).

Gender is often considered an unimportant variable in the evaluation of biomarkers of human disease; however, it is now clear that the severity of SARS-CoV-2 infection might be correlated with gender. Therefore, we explored whether metabolomics and lipidomics profiles were linked with gender; the PCA (Figure S10A–C) indicated that male and female patients were well distributed across all samples and that clusters specifically related to males or females were not present. Moreover, we did not find gender differences in the main circulating biomarkers (Figure S10D–G).

We further investigated and validated the diagnostic performances of these biomarkers by analyzing non-COVID-19 patients with similar symptoms as those with the COVID-19 infection. The analysis confirmed the validity of most of the selected lipidic biomarkers in detecting COVID-19 infection. Phosphatidylcholine 14:0_22:6, phosphatidylcholine 16:1_22:6, and phosphatidylethanolamine 18:1_20:4 showed AUC values of 0.89 (SE: 83%, SP: 84%), 0.89 (SE: 88%, SP: 80%), and 0.87 (SE: 83%, SP: 86%), respectively. The ROC curves for arachidonic acid (FA 20:4), oleic acid (FA 18:1), glycerophosphoethanolamines PE (O-18:2_20:4), and glycerophosphoethanolamines PE (O-16:1_18:2) were characterized by AUC values of 0.84 (SE: 74%, SP: 88%), 0.87 (SE: 81%, SP: 86%), 0.85 (SE: 83%, SP: 80%), and 0.82 (SE: 76%, SP: 81%), respectively (Figure S11). The diagnostic performances of the metabolites resulted in AUC values that ranged from 0.70 (2-hydroxy-3-methylbutyric acid) to 0.63 (butanedioic acid) (Figure S12). 

## 3. Discussion

The present study provides the first largest untargeted metabolomics and lipidomics analysis of plasma from COVID-19 patients and control groups. Patients were enrolled from a hospital located in northern Italy, the Italian epicenter of the pandemic. Italy has been one of the red hotspots for the COVID-19 and the first western country to experience the COVID-19 disease. 

Although the metabolome profile of COVID-19 patients has been investigated in other studies [14,15,16,18], these analyses were performed on small sets of samples, gender-specific analyses were not carried out [14,16,18], and, in one case, a young control population was used [18]. Moreover, the mean age of COVID-19-positive patients was 43 [16], 48 [15], and 56 [18] years, whereas elderly patients are at higher risk and have a higher mortality [24]. Our research involved a larger number of patients (*n* = 161) with respect to the previous study, distributed between critical and non-critical COVID-19 patients (*n* = 103), critical and non-critical patients admitted for pneumonia and/or respiratory failure with negative COVID-19 test results (*n* = 32), and healthy individuals (*n* = 26). The mean age of COVID-19 patients involved in our study was 67 years, the frailest population group.

**Figure 8:**
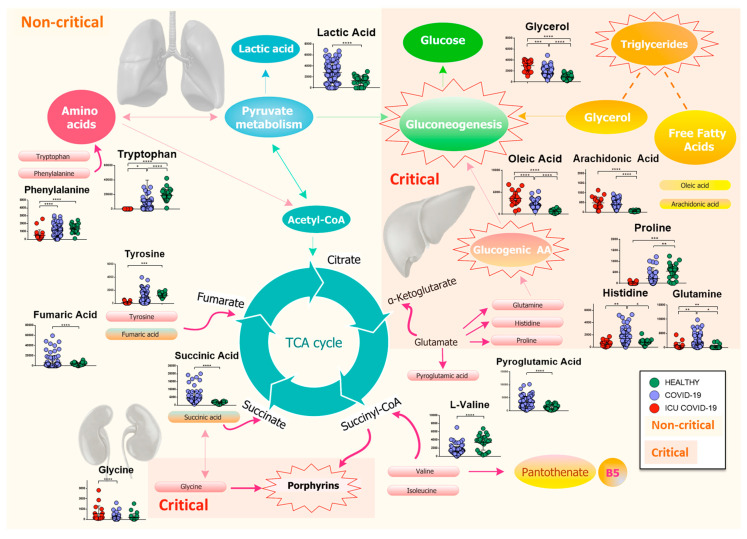
Mapping of differential metabolites related to the major metabolism involved during the progress of the COVID-19 infection. The biochemical map shows the presence of an increase in lactic acid and a consumption of amino acids, which enter the TCA cycle as intermediates of fumarate and succinyl-CoA, in non-critical patients. Isoleucine and l-valine may cause a dysregulation of pantothenate metabolism, resulting in a possible loss of vitamin B5. Meanwhile, pyroglutamic acid, which can be converted into glutamic acid and enter TCA for energy production, increases, as does succinic acid. The severity of the disease is characterized by a drastic decrease in glucogenic amino acids, which are used in the process of gluconeogenesis, while lipolysis of adipose tissue produces glycerol, which is used for the synthesis of glucose, and free fatty acids, such as arachidonic and oleic acids. Finally, the increase in glycine activates the metabolism of porphyrins, which play a crucial role in the progression of the infection. (*p*-value < 0.05 = *; *p*-value < 0.01 = **; *p*-value < 0.001 = ***; *p*-value < 0.0001 = ****).

Our findings show a slightly different host response respect to the study mentioned above, which may be due to the larger number of patients involved in our study, to the older population, and to the different geographic origin of patients. In particular, we found that the host response to the virus directly or indirectly involves several metabolisms, inflammation, immune system, and the global plasma lipidome. Figure 8 reports the map of differential metabolites related to the major metabolism involved during the progress of the COVID-19 infection.

**Figure 9:**
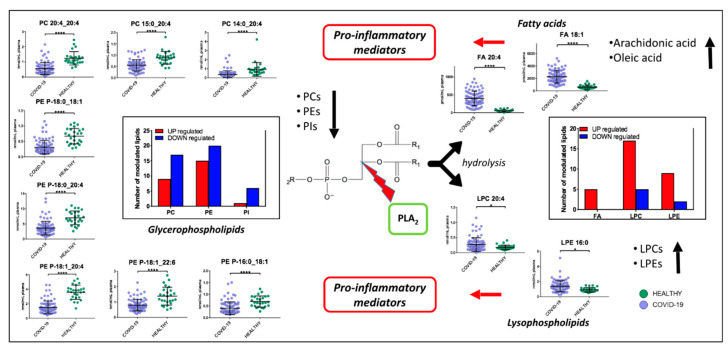
Proposed mechanism involved in COVID-19 pathogenesis. PLA2 hydrolyze phospholipids to yield fatty acids and lysophospholipids. We found a downregulation of glycerophospholipids (PCs, PEs and PIs) and upregulations of lysophospholipids (LPCs and LPEs), arachidonic acid, and oleic acid. Fatty acids and lysophospholipids are pro-inflammatory mediators. (*p*-value < 0.05 = *; *p*-value < 0.0001 = ****).

Non-critical patients were characterized by a strong alteration of lipids, including acylcarnitines, diacylglycerols, fatty acids, glycerophosphoethanolamines, glycerophosphocholines, sphingomyelins, and triacylglycerols. The infection seems to cause a global downregulation of glycerophospholipids. The significant roles of glycerophospholipids and fatty acids, such as arachidonic acid, in HCoV-229E-infected cells have already been identified; for example, the addition of arachidonic acid suppressed HCoV-229E and MERS-CoV replication [25]. High levels of fatty acids may be a defense mechanism of the host against the virus but at the same time may cause inflammation. It has already been shown that, in severe COVID-19 inflammation, arachidonic acid modulates the production of cytokines, causing a real cytokine storm [26,27]. On the other hand, it has also been demonstrated that cytokines induce the release of unsaturated fatty acids as a defense mechanism against invading microorganisms responsible for respiratory diseases [28]. Among glycerophospholipids, PCs, which are synthesized in the liver and are the only phospholipids necessary for lipoprotein, were the most downregulated the downregulation of this lipid class was already identified in COVID-19 patients and associated with hepatic impairments [14,16]. Additionally, decreases in PCs in blood plasma have been found in sepsis, cancer, and Dengue infection [29], while in COVID-19 patients Song et al. observed a decrease of PAs and PIs as well [16]. We observed that some lipids (Figure 9), such as PE (P-16:0_18:1), PE (P-18:1_22:6), PE (P-18:1_20:4), PE (P-18:0_20:4), PE (P-18:0_18:1), PC (20:4_20:4), PC (P-15:0_20:4), and PC (P-14:0_20:4) have one of the acyl chains constituted by C20:4 or C18:1, that are, the structure of the arachidonic acid and oleic acid, respectively, which can be oxidized to prostaglandins, well-known players in the inflammatory response [30]. Lipid mediators, such as oleic acid and docosahexaenoic acid (DHA), have been associated with the antiviral response in mouse models of influenza virus infection [31]. In addition, we found an upregulation of LPCs, which are a group of bioactive lipids heavily investigated in the context of inflammation. Higher lysophosphatidylethanolamine and lysophophatidylcholine levels can be linked to viral replication that can induce dramatic lipid changes, as already reported for the Dengue virus [29] and in COVID-19 [16]. Plasma LPCs are mainly produced by the action of phospholipases A2 (PLA2) after the removal of a fatty acid [32]. LPCs are widely regarded as potent pro-inflammatory mediators and their concentration can drastically increase in certain inflammatory states. Primarily, LPCs are recognized as important homeostatic mediators involved in all stages of vascular inflammation. In particular, LPC is able to produce a defect in endothelium-dependent vasomotor regulation, by reducing the production of nitric oxide (NO) and prostaglandin PGI2 in endothelial cells [33]. Moreover, LPCs may directly contribute to immune cell infiltration during vascular inflammation by increasing the expression of adhesion molecules such as intercellular adhesion molecule 1, vascular cell adhesion protein 1, and P-selectin [34], through the production of chemokines such as monocyte chemoattractant protein, IL-8, and Chemokine (C-C motif) ligand 5. Furthermore, LPC was found to act as a strong chemoattractant for monocytes [35], T cells [36], as well as natural killer (NK) cells [37], attracting them to sites of inflammation. Moreover, LPCs are demonstrated to potentiate the activation of T lymphocytes, while having no effect on resting cells [38]. ROS production and chemokine receptor expression in human Jurkat T cells are significantly increased upon LPC addition [39]. Furthermore, LPCs enhance IFN-secretion and gene expression in CD4+ and CD8+ T cells as well as increased CD40L and CXCR4 expression in CD4+ T cells [40]. Finally, studies performed on monocytes and macrophages show that LPCs are able to activate macrophages, increase their phagocytic activity in the presence of T lymphocytes [41], and enhance the oxidative burst and reactive oxygen production in neutrophils [42]. A dysregulation of macrophages was already identified in COVID-19 patients [15]. The downregulation of glycerophospholipids (PCs, PEs and PIs) and the upregulation of lysophospholipids (LPCs and LPEs), arachidonic acid, and oleic acid, would suggest a strong involvement of PLA2 in the pathogenesis and progression of COVID-19 (Figure 9). PLA2 is a group of enzymes that hydrolyze phospholipids to yield fatty acids and lysophospholipids. The reaction is known as the initial, rate-limiting step of arachidonate metabolism leading to the production of bioactive lipid mediators including prostaglandins, leukotrienes, and lysophospholipids [43]. PLA2 is critically involved in coronavirus replication, most likely by producing lysophospholipids that are required to form the specialized membrane compartments in which viral RNA synthesis takes place [44], while the analysis of SARS-CoV-2 infected host cell proteomics showed a potential link with inflammatory response, supported by the increase of PLA2 at 24 h after virus infection [45]. Our study provides further evidence for considering PLA2 activation as a potential key factor in the pathogenesis of COVID-19 and a potential therapeutic target.

Acylcarnitines, which were present in higher levels in infected patients, are secreted by mitochondria; it has already been reported that long-chain acylcarnitines may represent a risk factor for lung injury in humans with dysfunctional FAO since these lipids can accumulate at the air-fluid interface when metabolism is inhibited [46]. Respiratory viruses, such as the influenza virus, exacerbate acylcarnitine accumulation; the increase of acylcarnitines in the plasma of COVID-19 patients may be linked to this mechanism. Several studies have suggested that acylcarnitine accumulation may be toxic, particularly amphiphilic long-chain acylcarnitines, which have been shown to inhibit ion channels, disrupt calcium signaling, and impair ATP [47].

The identification of high levels of triacylglycerols suggests an increase in adipose tissue lipolysis in COVID-19 patients. The high levels of free fatty acids confirm the role of adipose tissue in the circulating lipid profile [22]. In particular, we also noted higher levels of TGs with longer fatty acid chains (an average of 54 carbons) and polyunsaturated (an average of 5 double bonds). Through the pathway analysis we confirmed a modulation of the glycerophospholipid metabolism pathway in COVID-19 patients, which was also identified by Wu et al. [14]. Likewise, an increase in serum C18-FFAs has already been associated with the development of acute respiratory distress syndrome [48]. We also observed an increase of FA 18:1 and FA 18:2 in COVID-19 patients. 

Non-critical patients were also characterized by altered metabolic pathways. The metabolism of amino acids, such as phenylalanine, tyrosine and tryptophan biosynthesis, phenylalanine metabolism, and aminoacyl-tRNA, suggested that this metabolism might play a major role in the response to the virus. The involvement of the biosynthesis of the unsaturated fatty acid pathway may contribute to the upregulation of arachidonic, oleic, palmitic, and stearic acids in infected patients. 

The downregulation of the main amino acids involved in the stability of aa-tRNA suggests that COVID-19 infection can lead to their rapid consumption, as confirmed by Wu et al. [14]. In particular, we found the downregulation of histidine, l-valine, l-proline, and tryptophan in patients with COVID-19. Moreover, our findings included higher levels of lactic acid, which may be caused by the tissue hypoxia that commonly occurs in this viral infection or other severe infections [13,49]. This increase could also be directly correlated to the immune system response [50]. Downregulated l-valine can be considered a potential marker of the infection, and its further involvement in the dysregulation of pantothenate and CoA biosynthesis, which is the pathway responsible for the production of pantothenic acid, could cause a lack of vitamin B5 and compromise the mitochondrial energy metabolism [51], as confirmed by the enrichment analysis (Figure 5B).

However, the levels of fumaric and succinic acids, which are intermediates of the TCA cycle, were reduced and increased, respectively. In addition, the level of pyroglutamic acid, which can be converted into glutamic acid and enter the TCA cycle for further energy production or substance synthesis, was increased in infected patients. A general reduction of amino acids involved in TCA cycle was identified in COVID-19 [16].

Another important finding of this study is the involvement of butanoate metabolism. As already demonstrated by Chemudupatiet et al. [52], high levels of the short chain fatty acid butyrate increase cellular infection and promote virus replication. The upregulation of butyric acid and derivatives in the plasma of COVID-19 patients could explain this correlation.

Regarding critical patients, triglycerides were strongly upregulated. Adipose tissue is important in adapting to critical illness; in fact, during starvation, the lipolysis of adipose tissue increases [53], not only converting TG to free fatty acids but also resulting in the enhanced recycling of the fatty acids back into TGs, as supported by our results and already reported in relation to EBOV infection [22].

Pathway analysis also identified a dysregulation of porphyrin metabolism, since the levels of glycine, which is a precursor in the first step of production of porphyrin [54], were shown to be upregulated in ICU-COVID-19 patients. It has recently been shown that the increased viral proteins and decreased hemoglobin in severe patients lead to the formation of porphyrin, which plays a very important role in the progress of the COVID-19 infection [55]. This activity of SARS-CoV-2 on hemoglobin has been considered a possible basic pathogenic mechanism [56], along with the hypothesis that COVID-19 is an acquired acute porphyria [57]. The association of porphyrin to COVID-19 confirmed that this infection not only involves the lungs (via pneumonia) but also other organs. Abrahams et al. [57] showed that severe COVID-19 patients experienced a form of acquired acute porphyria; readily available interventions exist to treat this disease, while urinalysis could be used for the diagnosis of this pathology. The potential involvement of iron homeostasis in COVID-19 infection was already suggested [58]; our COVID-19 patients were characterized by high levels of ferritin, as already observed in other studies [16,59,60]. Despite the fact that critical COVID-19 patients were all treated with hydroxychloroquine, we cannot exclude the possibility that the involvement of this pathway was caused by the effect of the drug. In fact, chloroquine and hydroxychloroquine have been shown to modulate iron metabolism, compromising its homeostasis at different levels and reducing inflammatory cytokines [61], even if on COVID-19 patients the real effect of this drugs is still to be clarified. In many virus infections, iron overload is associated with poor prognosis and could be partly caused by the viruses themselves [62]. According to recent literature, chloroquine and hydroxychloroquine should be avoided in patients with porphyria [63], while a careful monitoring of liver and kidney functions in COVID-19 patients should always be performed [64]. In addition, a recent study suggests avoiding hydroxychloroquine in patients admitted to hospital with COVID-19 who require oxygen [65].

Our results clearly demonstrates the involvement of hepatic and renal functions in critical patients through the porphyrin metabolism.

The comparison between non-critical and critical patients confirmed the direct correlation of an abundance of TGs with the severity of the disease, while the glycerophosphocholine downregulation was consistent with our data reported above on non-critical COVID-19 patients. SMs, which were downregulated in critical patients, as already found in COVID-19 patients [16], are a type of sphingosine-containing phospholipid that is synthesized by the transfer of a phosphocholine residue from a phosphatidylcholine to a ceramide. Together with glycerophospholipids, sphingolipids are important components of membrane cells and regulate several processes, such as growth regulation and inflammatory responses [13]. A reduced level of SMs impairs the infection and limits the virus entry [66]. Therefore, SM metabolism can be a potential target for therapeutic intervention against COVID-19 virus infection.

Finally, the upregulation of gluconeogenesis could be associated not only to the strong degradation of glucogenic amino acids (GAAs), such as phenylalanine, histidine, proline, and tryptophan, which are probably used by gluconeogenesis to produce glucose, but also to the increased levels of triglycerides and glycerol (from the breakdowns of lipids) in ICU-COVID-19 patients. GAAs in severe patients were strongly downregulated compared to non-critical patients. The levels of GAAs reflect the severity of the disease and it is not a consequence of the treatment received in ICU. In fact, as shown in Figure S13, there is a significant downregulation of proline, histidine, and glutamine in ICU-COVID-19 patients respect with ICU non-COVID-19.

We also investigated the potential role of lipids and small molecules as biomarkers for the COVID-19 infection. Arachidonic acid and oleic acid were the best biomarkers with AUC values of 0.99 and 0.98, respectively, when compared to healthy subjects, while the addition of non-COVID-19 patients with similar symptoms dropped the AUC values to 0.87 and 0.84, respectively. 

An increase of fatty acids abundance was already found in COVID-19 patients [15,16], although they were not a good candidate as biomarkers. Interestingly, the analysis of non-COVID-19 patients with similar symptoms as COVID-19 patients confirmed the validity of lipid biomarkers, suggesting that lipids, especially our reported molecules, are directly linked to the host response to the virus, while small molecules are probably more sensitive to other disease states. Moreover, our data shows that oleic acid and arachidonic acid levels are directly correlated to the severity of the disease (Figure S6). In addition, we found no statistical differences between ICU-COVID-19 and COVID-19 compared to ICU non-COVID-19 patients (Figure S14). Therefore, we can assume that the ICU treatments do not affect the correlation with the severity in COVID-19. On the other hand, these two biomarkers are not suitable to distinguish COVID-19 patients from ICU non-COVID-19 patients.

In addition, several circulating lipids acted as biomarkers, such as phosphatidylcholine 14:0_22:6 (AUC = 0.96), phosphatidylcholine 16:1_22:6 (AUC = 0.97), and phosphatidylethanolamine 18:1_20:4 (AUC = 0.94). While other research studies on COVID-19 were able to identify infected patients using multiple metabolites, i.e., Song et al. proposed a panel of 10 molecules, here we propose the use of single biomarkers. At the same time, by combining more molecules the accuracy and sensitivity of the diagnosis may increase. 

Taken together, our study provides evidence that lipids and metabolic dysfunction are strongly involved in COVID-19. These results offer crucial evidence in relation to the potential involvement of PLA2 in the SARS-CoV-2 pathogenesis, which may thus represent a candidate target for antiviral drug development. Future studies should examine the levels and activity of PLA2 and the provenance. Most importantly, these findings may improve the understanding of SARS-CoV-2-induced metabolic alterations and may aid in the development of the most appropriate therapies and improved measures to control the spread of the virus.

Although, to date, this is the largest untargeted LC-MS/MS and GCxGC-MS research study in the literature, the average age of COVID-19 patients in our cohort is higher than the healthy and negative subjects used as controls. Moreover, the addition of asymptomatic COVID-19 infected patients in the study would have allowed for the identification of more specific pathways and molecules associated with the development of this disease.

## 4. Materials and Methods

### 4.1. Patients

Plasma samples from 161 subjects, admitted to Novara University Hospital for pneumonia and/or respiratory failure from March to April 2020, were collected at the Emergency Department or at COVID-19 wards including the Intensive Care Unit. Of these, 103 had a confirmed diagnosis of SARS-CoV-2 infection by reverse-transcriptase polymerase chain reaction (RT-PCR).

We considered critical patients those with respiratory failure admitted to the intensive care unit requiring mechanical ventilation (ICU-COVID-19), while non-critical patients all other patients with mild to severe respiratory failure requiring oxygen supplementation but neither mechanical nor non-invasive ventilation. Clinical characteristic of the patients are reported in Table 1.

Out of the 103 COVID-19 patients enrolled, 19 were critical and 84 non-critical. Out 34 patients admitted for pneumonia and/or respiratory failure with negative nucleic acid test results, 20 were non-critical and 12 critical. Healthy individuals (*n* = 26) were enrolled as controls. The Institutional Review Board (Comitato Etico Interaziendale Novara) approved this study (n. RQ06320/25 March 2020).

### 4.2. Materials and Reagents

For the analysis, LC-MS-grade solvents and reagents were used. Formic acid, ammonium formate, tert-Butyl methyl ether, BSTFA, methoxyamine, Hexadecane, and tridecanoic acid were procured from Merck (Darmstad, Germany), pyridine, water and acetonitrile were from VWR (Milano, Italy), and methanol and isopropanol were from Scharlab (Barcelona, Spain). As internal standards for lipidomics analysis we employed the SPLASH Lipidomix^®^ [PC 15:0-18:1(d7); PE 15:0-18:1(d7); PS 15:0-18:1(d7); PG 15:0-18:1(d7); PG 15:0-18:1(d7); PA 15:0-18:1(d7); LysoPC 18:1(d7); LysoPE 18:1(d7); Chol Ester 18:1 (d7); 18:1(d7) MG; DG 15:0-18:1(d7); TG 15:0-18:1(d7)-15:0; SM 18:1(d9); Cholesterol (d7)]; DG 12:0-12:0 (Avanti Polar Lipids, Alabaster, AL, USA); and 12-[[(cyclohexylamino)carbonyl]amino]-dodecanoic acid (CUDA) (Cayman Chemicals, Ann Arbor, MI, USA). 

### 4.3. Sample Preparation for Metabolomics Analysis

To prepare the samples, 1 mL of an ACN/IPA/water (3:3:2) solution, with tridecanoic acid at 1 ppm as internal standard, was added to 30 µL of plasma. After vortexing, the sample was centrifuged at room temperature for 15 min at 14,500× *g*. The supernatant was then dried in a speed-vacuum. The sample was derivatized with methoximation (20 µL of Methoaxime, 80 °C, 20 min) and underwent sialylation (90 µL of BSTFA, 80 °C, 20 min). After this, 10 µL of hexadecane (IS) were added and the sample was ready for the GC-MS analysis. 

### 4.4. GCxGC/TOFMS Analysis

For metabolomics analysis, a LECO Pegasus BT 4D GCXGC/TOFMS instrument (Leco Corp., St. Josef, MI, USA) equipped with a LECO dual stage quad jet thermal modulator was used. The GC part of the instrument was an Agilent 7890 gas chromatograph (Agilent Technologies, Palo Alto, CA, USA), equipped with a split/splitless injector. The first dimension column was a 30 m Rxi-5Sil (Restek Corp., Bellefonte, PA, USA) MS capillary column with an internal diameter of 0.25 mm and a stationary phase film thickness of 0.25 μm, and the second dimension chromatographic column was a 2 m Rxi-17Sil MS (Restek Corp., Bellefonte, PA, USA) with a diameter of 0.25 mm and a film thickness of 0.25 μm. High-purity helium (99.9999%) was used as the carrier gas with a flow rate of 1.4 mL/min. Then, 1 μL of sample was injected in splitless mode at 250 °C. The temperature program was as follow: the initial temperature was 70 °C for 2 min, then ramped 6 °C/min up to 160 °C, 10 °C/min up to 240 °C, 20 °C/min to 300, and then held at this value for 6 min. The secondary column was maintained at +5 °C relative to the GC oven temperature of the first column. The programming rate was the same for the two columns. Electron impact ionization was applied (70 eV). The ion source temperature was set at 250 °C, the mass range was 25 to 550 *m*/*z* with an extraction frequency of 32 kHz for the bi-dimensional analysis and 30 kHz for mono-dimensional. The acquisition rates were 200 spectra/s for 2D analysis. The modulation period for the bi-dimensional analysis was 4 s for the entire run. The modulator temperature offset was set at +15 °C relative to the secondary oven temperature, while the transfer line was set at 280 °C.

### 4.5. Metabolomics Data Analysis

The chromatograms were acquired in TIC (total ion current) mode. Peaks with signal-to-noise (S/N) value lower than 500.0 were rejected. ChromaTOF version 5.31 was use for the raw data processing. Mass spectral assignment was performed by matching with NIST MS Search 2.3 libraries and the FiehnLib. An in-house library of standards was also used for small molecules identification. Statistical analysis was performed with Metaboanalyst software (www.metaboanalyst.org) [67].

### 4.6. Quality Control of Metabolomics Analysis

Quality control procedures were included in the experiment and during the analysis. Pooled samples were prepared using some patients’ plasma and used for system suitability tests. The pool samples were run before using the instrument for patients’ samples, at the beginning of the batch and at the end of the batch. Blanks were run at the beginning and at the end of the batch to check for residual interference. Internal standards (tridecanoic acid and hexadecane) were spiked in each sample and used for instrument stability monitoring and/or data normalization. Instrument variability was determined by calculating the coefficient of variation percentage (CV%) of internal standards in each sample and of endogenous metabolites present in the pooled quality control samples. 

### 4.7. Sample Preparation for Lipidomics Analysis

The extraction of plasma lipids was carried out using a biphasic method [68]: 30 µL of plasma were placed in a tube and extracted with 225 µL of cold MeOH, containing a mix of deuterated standard (Splash Lipidomix^®^). Then, the solution was vortexed for 10 s, followed by the addition of 750 µL of cold MTBE and vortexed for 10 s. The tube was then placed in a thermomixer at 4 °C and shacked for 6 min at 2000 rpm. After this, 188 µL of water were added and the tube was vortexed for 10 s and then centrifuged for 2 min at 14,000 rpm at 4 °C. Finally, 300 µL of supernatant was collected and evaporated using a SpeedVac.

The dried sample was reconstituted with 50 µL of a solution MeOH/Toluene 9:1 containing the internal standard CUDA (12.5 ng/mL).

### 4.8. LC-MS/MS Analysis 

The reconstituted samples were analyzed by an UHPLC Vanquish system (Thermo Scientific, Rodano, Italy) coupled with an Orbitrap Q-Exactive Plus (Thermo Scientific, Rodano, Italy). The separation of lipids was achieved by a reverse phase column (Hypersil Gold™ 150 × 2.1 mm, particle size 1.9 µm), the column was maintained at 45 °C at a flow rate of 0.260 mL/min. Mobile phase Afor the ESI positive mode consisted of acetonitrile/water 60:40 (*v*/*v*) with ammonium formate (10 mmol) and 0.1% formic acid while B was isopropanol/acetonitrile 90:10 (*v*/*v*) with ammonium formate (10 mmol) and 0.1% formic acid, while in the negative ESI mode the organic solvents for both mobile phases were the same as positive with the exception of using ammonium acetate (10 mmol) as mobile-phase modifier. The gradient used was as follows: 0–2 min from 30% to 43% B, 2–2.1 min from 43% to 55% B, 2.1–12 min from 55% to 65% B, 12–18 min from 65% to 85% B, 18–20 min from 85% to 100% B; 100% B was kept for 5 min and then the column was allowed to re-equilibrate at 30% B for another 5 min. The total run time was 30 min.

Mass spectrometry analysis was performed in both positive and negative ion mode. The source volt­age was maintained at 3.5 kV in the positive ion mode and 2.8 kV in the negative ion mode. All other interface settings were identical for the two types of analysis. The capillary temperature, sheath gas flow, and aux­iliary gas flow were set at 320 °C, 40 arb, and 3 arb respectively. S-lens was settled at 50 rf. Data were collected in a data-dependent (ddMS2) top 10 scan mode. Survey full-scan MS spectra (mass range *m*/*z* 80 to 1200) were acquired with resolution R = 70,000 and AGC target 1 × 10^6^. MS/MS fragmentation was performed using high-energy c-trap dissociation (HCD) with resolution R = 17,500 and AGC target 1 × 10^5^ The stepped normalized collision energy (NCE) was set to 15, 30, and 45, respectively. The injection volume was 3 µL. Lockmass and regular inter-run calibrations were used for accurate mass-based analysis. An exclusion list for background ions was generated analyzing the same procedural blank sample, both for the positive and negative ESI mode.

### 4.9. Lipidomics Data Processing

The acquired raw data from the untargeted analysis were processed using MSDIAL software (Yokohama City, Kanagawa, Japan), version 4.24 [20]. This included the detection of peaks, MS2 data deconvolution, compound identification, and the alignment of peaks through all the samples. For identification a cut off value of 85% was selected: this value is based on 6 different similarity scores: 1 for retention time, 1 for *m*/*z* 1 for isotopic pattern, and 3 for MS/MS (dot product, dot product reversed and presence). Peaks corresponding to internal standards were removed from MS-Dial detected features and were analyzed in Skyline program to evaluate the reproducibility. The dataset containing *m*/*z* values, retention time, peak area, and annotation from the aligned files were exported as an Excel file and manually checked in order to eliminate signals from blanks or wrong records. For quantification, the peak area for different detected molecular species for each particular lipid was combined (e.g., [M + NH_4_]+ & [M + Na]+ for TG) followed by normalization using the deuterated internal standard for each lipid class. In order to obtain an estimated concentration expressed in nmol/mL (plasma) the normalized areas were multiplied by the concentration of the internal standard. An in-house library of standards was also used for lipids identification.

MetaboAnalyst 4.0 software (www.metaboanalyst.org) was used for statistical analysis while Lipea software (https://lipea.biotec.tu-dresden.de/home) was used for pathway analysis [69]. The data provided in this article have been deposited to the EMBL-EBI MetaboLights database with the identifier MTBLS1866.

### 4.10. Quality Control of Lipidomics Analysis

The stability of retention time, mass accuracy, and intensity are essential in LC-MS based lipidomics analysis. Quality control was assured by analyzing pooled samples before the batch, at the beginning of the batch, and at the end of the batch; injecting blanks to check for residual interference; by using internal standards, directly in the plasma samples that cover a number of analyte classes at appropriate levels for plasma (Avanti SPLASH Lipidomix), and an internal standard (CUDA) before the LC-MS analysis. Since the analysis were performed over a long time period, the pool samples were made using plasma from subjects not included in this study since we wanted to preserve the quality of patients’ samples and avoid unnecessary freeze–thaw cycles. Instrument variability was determined by calculating the coefficient of variation percentage (CV%) of internal standards in each sample and in the pooled quality control samples.

## Figures and Tables

**Table 1:** Characteristics of the patients included in the study.

Variable	Non-COVID-19 Patients				COVID-19 Patients		
	Total (58)	Healthy Control (*n* = 26)	Non-critical (*n* = 20)	Critical (*n* = 12)	Total (*n* = 103)	Non-critical (*n* = 84)	Critical (*n* = 19)
**Sex (no.)**							
Male	23	11	9	6	61	48	13
Female	29	15	11	6	42	36	6
**Age (year)**							
Mean ± SD	61.8 ± 15.4	50.1 ± 5.3	68.6 ± 8.9	67.4 ± 17.3	67.3 ± 18.0	59.7 ± 13.0	69.0 ± 18.5
Range	38.0–96.0	42.0–56.0	56.0–82.0	38.0–96.0	21.0–107.0	21.0–76.0	29.0–107.0
**Time from onset to admission (days)**							
Mean ± SD			5.7 ± 10.0	7.7 ± 6.5	5.8 ± 7.2	5.8 ± 7.6	5.5 ± 5.0
Range			1.0–45.0	1.0–12.0	1.0–32.0	1.0–32.0	1.0–19.0
**Time from admission to severe (days)**							
Mean ± SD				1.8 ± 4.9			6.5 ± 7.3
Range				1.0–13.0			1.0–28.0
**Symptoms (n° of patients)**							
Fever			9	0	52	40	12
Cough			5	0	34	25	13
Headache			0	0	1	1	0
Fatigue			1	1	8	8	0
Dyspnea			4	0	27	23	4
Diarrhea			2	1	13	9	4
Chest pain			3	0	5	5	0
Abdominal pain			4	0	5	4	1
Vomiting			6	0	3	3	0
**Comorbidity (n°)**							
Hypertension			0	2	38	29	9
Diabetes			0	1	17	12	5
Respiratory system			1	0	6	6	0
Cardiovascular system			4	1	38	34	4
Other endocrine system			0	0	12	9	3
Chronic kidney			1	0	9	7	2
Digestive system			2	0	16	15	1
**Oxygen saturation index (%)**							
Mean ± SD			85.5 ± 6.3	94.3 ± 3.8	90.7 ± 6.7	90.8 ± 6.4	90.3 ± 8.2
Range			81.0–90.0	87.0–99.0	71.0–99.0	71.0–99.0	71.0–98.0

## Supplementary Materials

The following are available online at https://www.mdpi.com/1422-0067/21/22/8623/s1, Figure S1. Principal component analysis score plot of COVID-19 patients (red dots), healthy subjects (green dots) and QC samples (green dots). Figure S2. Principal component analysis (PCA) score plot of non-critical positive patients (COVID-19) (red dots) and healthy subjects (green dots). Figure S3: Partial Least Square Discriminant Analysis (PLS-DA) of non-critical positive patients (COVID-19) (red dots) and healthy subjects (green dots). Figure S4: Heat-maps of top modulated lipids in positive (A) and negative (B) mode and of small molecules (C) for the comparison between ICU-COVID-19 versus ICU. Principal component analysis of ICU-COVID-19 and ICU patients. The graphs show a clear separation between the two classes of samples. Figure S5: Modulated lipid classes (A) and number of up-regulated and down-regulated lipids (B) in ICU-COVID-19 patients respect with ICU. Figure S6: Bar plots (average ± SD) of FA 20:4 (A) and FA 18:1 (B) in healthy subjects, non-critical and severe COVID-19 patients. Figure S7: Metabolic pathway analysis (left) performed on modulated metabolites and metabolite sets enrichment (right) in ICU-COVID-19 versus ICU. Figure S8: Modulated lipid classes (A) and number of up-regulated and down-regulated lipids (B) in COVID-19 patients respect with ICU-COVID-19 patients. Figure S9: Metabolic pathway analysis (A) performed on modulated metabolites and metabolite sets enrichment (B) in COVID-19 versus ICU-COVID-19. Figure S10: Score plots from PCA indicating COVID-19 female patients (red dots), COVID-19 male patients (green dots), healthy female subjects (violet dots) and healthy male patients (blue dots). Figure S11: Validation of selected potential lipid biomarkers including non-COVID-19 patients with similar symptoms as those with the COVID-19 infection. Figure S12: Validation of selected potential molecules biomarkers including non-COVID-19 patients with similar symptoms as those with the COVID-19 infection. Figure S13: Bar plots (average ± SD) of histidine (A), glutamine (B) and proline (C) in healthy subjects, non-critical and severe COVID-19 patients and in critical non-COVID-19 patients. Figure S14: Bar plots (average ± SD) of FA 18:1 (left) and FA 20:4 (right) in healthy subjects, non-critical and severe COVID-19 patients and in critical non-COVID-19 patients. Table S1. Identified, quantified and modulated lipids. Table S2. RSDs of internal standards in QC samples of lipidomics analysis. Table S3. Identified, quantified and modulated small molecules. Table S4. RSDs of internal standards in QC samples of metabolomics analysis.
