# Supplementary material for: Large-Scale Plasma Analysis Revealed New Mechanisms and Molecules Associated with the Host Response to SARS-CoV-2
Source: Int J Mol Sci. 2020 Nov 16;21(22):8623. doi: 10.3390/ijms21228623 (PMC7696386; doi:10.3390/ijms21228623)
Supplement: Supplementary file 1 [file ijms-21-08623-s001.zip › supplementary material.doc]

Large-scale plasma analysis revealed new mechanisms and molecules associated with the host response to SARS-CoV-2

**Authors:** Elettra Barberis^1,2^, Sara Timo^2,3^, Elia Amede^1,2^, Virginia V. Vanella^1,2^, Chiara Puricelli^4^, Giuseppe Cappellano^4,2^, Davide Raineri^4,2^, Micol G. Cittone^5,6^, Eleonora Rizzi^5,6^, Anita R. Pedrinelli^5,6^, Veronica Vassia^5,6^, Francesco G. Casciaro^5,6^, Simona Priora^5,6^, Ilaria Nerici^5,6^, Alessandra Galbiati^5,6^, Eyal Hayden^5,6^, Marco Falasca^7^, Rosanna Vaschetto^1^, Pier Paolo Sainaghi^5,6^, Umberto Dianzani^4^, Roberta Rolla^4^, Annalisa Chiocchetti^4,2^, Gianluca Baldanzi^1,2^, Emilio Marengo^2,3^, Marcello Manfredi^1,2^*

**Affiliations:**

^1^Department of Translational Medicine, University of Piemonte Orientale, Novara, Italy.

^2^Center for Translational Research on Autoimmune and Allergic Diseases, University of Piemonte Orientale, Novara, Italy.

^3^Department of Sciences and Technological Innovation, University of Piemonte Orientale, Alessandria, Italy.

^4^Department of Health Sciences, University of Piemonte Orientale, Novara, Italy.

^5^Internal and Emergency Medicine Departments, Department of Translational Medicine, University of Piemonte Orientale, Novara, Italy.

^6^Azienda Ospedaliero-Universitaria "Maggiore della Carità", Novara, Italy.

^7^Metabolic Signalling Group, School of Pharmacy & Biomedical Sciences, Curtin University, Perth, Australia.

*To whom correspondence should be addressed: [marcello.manfredi@uniupo.it](mailto:marcello.manfredi@uniupo.it)

**Supplementary Materials**

Fig. S1. Principal component analysis score plot of COVID-19 patients (red dots), healthy subjects (green dots) and QC samples (green dots). The analysis shows that QC replicates are well clustered together, reflecting the instrumental reproducibility for lipidomics in both positive (**A**) and negative (**B**) mode and for metabolomics (**C**).

Fig. S2. Principal component analysis (PCA) score plot of non-critical positive patients (COVID-19) (red dots) and healthy subjects (green dots). The two groups are well separated for positive mode (**A**) and negative mode (**B**) lipidomics analysis but also for metabolomics (**C**).

Fig. S3: Partial Least Square Discriminant Analysis (PLS-DA) of non-critical positive patients (COVID-19) (red dots) and healthy subjects (green dots). The two groups are well separated for positive mode (**A**) and negative mode (**B**) lipidomics analysis but also for metabolomics (**C**). Important features identified by PLS-DA for samples analyzed in positive mode (**D**) and negative mode (**E**) lipidomics analysis and metabolomics (**F**): colored boxes indicate the relative concentrations of the corresponding metabolite in each group under current study (red, up-regulation; green, down-regulation).

Fig. S4: Heat-maps of top modulated lipids in positive (**A**) and negative (**B**) mode and of small molecules (**C**) for the comparison between ICU-COVID-19 versus ICU. Principal component analysis of ICU-COVID-19 and ICU patients. The graphs show a clear separation between the two classes of samples.

Fig. S5: Modulated lipid classes (**A**) and number of up-regulated and down-regulated lipids (**B**) in ICU-COVID-19 patients respect with ICU.

Fig. S6: Bar plots (average ± SD) of FA 20:4 (**A**) and FA 18:1 (**B**) in healthy subjects, non-critical and severe COVID-19 patients.

Fig. S7: Metabolic pathway analysis (**left**) performed on modulated metabolites and metabolite sets enrichment (**right**) in ICU-COVID-19 versus ICU.

Fig. S8: Modulated lipid classes (**A**) and number of up-regulated and down-regulated lipids (**B**) in COVID-19 patients respect with ICU-COVID-19 patients.

Fig. S9: Metabolic pathway analysis (**A**) performed on modulated metabolites and metabolite sets enrichment (**B**) in COVID-19 versus ICU-COVID-19.

Fig. S10: Score plots from PCA indicating COVID-19 female patients (red dots), COVID-19 male patients (green dots), healthy female subjects (violet dots) and healthy male patients (blue dots). PCA was performed for metabolomics (**A**) and lipidomics in positive (**B**) and negative (**C**) mode. Male and female patients resulted well distributed across all samples and clusters specifically related to males or females were not present. Box-plots (average ± SD) of main circulating biomarkers FA 20:4 (**D**), FA 18:1 (**E**), 2-hydroxy-3-methylbutyric acid (**F**) and 2,3,4-trihydroxybutyric acid (**G**) with COVID-19 patients and healthy subjects divided for gender.

Fig. S11: Validation of selected potential lipid biomarkers including non-COVID-19 patients with similar symptoms as those with the COVID-19 infection. The figure reports the box-plots (average ± SD) and ROCs of Phosphatidylcholine 14:0_22:6 (**A**), phosphatidylcholine 16:1_22:6 (**B**), phosphatidylethanolamine 18:1_20:4 (**C**), glycerophosphoethanolamines PE (O-18:2_20:4) (**D**), glycerophosphoethanolamines PE (O-16:1_18:2) (**E**), arachidonic acid (FA 20:4) (**F**) and oleic acid (FA 18:1) (**G**).

Fig. S12: Validation of selected potential molecules biomarkers including non-COVID-19 patients with similar symptoms as those with the COVID-19 infection. The figure reports the box-plots (average ± SD) and ROCs of 2-hydroxy-3-methylbutyric acid (**A**), 2,3,4-trihydroxybutyric acid (**B**), 2-hydroxybutyric acid (**C**), 3-hydroxyisovaleric acid (**D**), palmitic acid (**E**), galactopyranose (**F**), L-pyroglutammic acid (**G**), butanedioic acid (**H**), and L-valine (**I**).

Fig. S13: Bar plots (average ± SD) of histidine (**A**), glutamine (**B**) and proline (**C**) in healthy subjects, non-critical and severe COVID-19 patients and in critical non-COVID-19 patients.

Fig. S14: Bar plots (average ± SD) of FA 18:1 (**left**) and FA 20:4 (**right**) in healthy subjects, non-critical and severe COVID-19 patients and in critical non-COVID-19 patients.

Table S1. Identified, quantified and modulated lipids.

Table S2. RSDs of internal standards in QC samples of lipidomics analysis

Table S3. Identified, quantified and modulated small molecules

Table S4. RSDs of internal standards in QC samples of metabolomics analysis
